# Gendered asymmetry of access to knowledge for brucellosis control among pastoral communities in north-west Côte d’Ivoire

**DOI:** 10.1186/s13570-022-00241-9

**Published:** 2022-06-23

**Authors:** Stephane A. Y. Babo, Gilbert Fokou, Richard B. Yapi, Coletha Mathew, Arnaud K. Dayoro, Rudovick R. Kazwala, Bassirou Bonfoh

**Affiliations:** 1 Université Félix Houphouët Boigny, Abidjan, Côte d’Ivoire; 2 Centre Suisse de Recherches Scientifiques en Côte d’Ivoire, Abidjan, Côte d’Ivoire; 3 Human Sciences Research Council, Cape Town, South Africa; 4 Centre d’Entomologie Médicale et Vétérinaire, Université Alassane Ouattara, Bouaké, Côte d’Ivoire; 5 Sokoine University of Agriculture, Morogoro, Tanzania

**Keywords:** Knowledge production, One Health, dairy, milk, Zoonosis

## Abstract

Brucellosis is an infectious zoonotic disease considered as a threat to public health and pastoralist livelihoods. Symptoms of the disease can lead to gender-specific ailments such as abortions in women and orchitis in men. Pastoralists and their families are at high risk of contracting the disease. Access to health information reinforces existing knowledge and contributes to disease prevention. However, in developing countries, interventions for knowledge sharing on zoonotic diseases predominantly target men. This study aimed to describe mechanisms of knowledge production and transfer on brucellosis according to gender, by assessing the way knowledge affects behaviours of pastoral communities. A community-based cross-sectional survey was conducted among a pastoral community (PC) of the Folon region in north-west Côte d’Ivoire. The study included transhumant pastoralists, sedentary livestock owners, shepherds and their wives. By using mixed methods, 26 semi-structured interviews were conducted, and 320 questionnaires were completed. Statistical analysis with chi-square (*χ*^2^) comparison tests was performed to compare variables between men and women. Findings were interpreted through the concept of specialisation of the social exclusion theory. We found that gender influences access to information on brucellosis and transfer of knowledge on brucellosis appeared gender-biased, especially from veterinarians towards men in the community. The social labour division and interventions of veterinarians through awareness reinforce the knowledge gap on brucellosis between men and women. Men and women consume raw milk, whilst only men in general handle animal discharges with bare hands. To improve the control of brucellosis, knowledge on best practice should be shared with pastoral communities using the One Health approach that encourages mutual learning. Innovative strategies based on gender daily tasks such as safe dairy processing by women and safe animal husbandry to expand their herd for men can be the entry point for the prevention of brucellosis.

## Introduction

Livestock diseases are a priority problem for livestock keepers throughout the world (Perry et al. 2001; Sargison 2020). Brucellosis appears as one of the most common infectious zoonotic diseases considered as a threat to public health and pastoralist livelihoods (Akakpo et al. 2010; Saegerman et al. 2014). In infected animals, brucellosis may cause an increase in abortion rate, temporary infertility, hygromas, orchitis and a drop in milk production (Mangen et al. 2002). In humans, raw milk consumption and unsafe handling or discharge of wastes from infected animals are common risk factors associated to the transmission of the disease (Hundal et al. 2016). In order to reduce disease occurrence, it is critical to understand how different involved stakeholders know about the disease (Abramowitz et al. 2015; Chenais et al. 2021). Information is an important component of behaviour change in the health system around the world (Brabin et al. 2010; Bieri et al. 2012). Lack of knowledge about brucellosis may affect the health-seeking behaviour of patients, thus leading to sustained transmission in these communities (Kansiime et al. 2014). Pathways of intervention and response to a disease are selectively justified by the various narratives constructed and mobilised about that disease (Leach and Tadros 2014). In animal health, whilst some pastoralists rely on the knowledge from veterinary services in case of animal diseases, others will only use traditional methods and self-administered treatments (Chenais et al. 2021). For both humans and animals, health interventions are affected by roles of societal structures such as the agency associated with age, marital or social status, and gender (Gammino et al. 2020). Asymmetry in access to information is a critical bottleneck faced by pastoral communities in their response to zoonotic diseases.

Gender-specific considerations are an important aspect to consider when dealing with access to healthcare. There are a number of factors that uniquely affect each gender related to both underlying biology (including, but not exclusively, their respective reproductive characteristics) and health education delivery and access (Doyal 2001; Davidson et al. 2006). However, in sub-Saharan Africa, gender disparities in health occur where women and young girls face a lack of access to health information and the power to decide for themselves when to seek healthcare (Pons-Duran et al. 2019; George et al. 2020). In some pastoral systems, women have less access to information on disease prevention, especially regarding zoonosis, when compared to men (Oluka et al. 2005; Mupawaenda et al. 2009; Njuki and Sanginga 2013). As a zoonotic disease, brucellosis affects both men and women (D’anastasio et al. 2011; Oguz and Oztek-Celebi 2018), with animals as the main source and reservoir of the disease (Godfroid et al. 2011). Common symptoms of brucellosis in humans are undulant fever, weakness, joint pains and arthritis (Zhe Liu et al. 2020). People living and working close to animals (shepherds, livestock owners and their families) are considered the most at risk of getting infected with the disease (Esmaeili et al. 2014). Thus, women constitute one of the most exposed social groups to brucellosis, whilst their access to information on the disease is limited. Lack of knowledge and perceptions on febrile illness such as brucellosis leads to self-medication which can impede a timely and full recovery (Mburu et al. 2021b).

In a context of national prioritisation of brucellosis as a disease to control in Côte d’Ivoire (Centers for Disease Control and Prevention and USAID 2017), assessing the knowledge of at-risk people, such as pastoral communities, is critical. Addressing the gendered knowledge asymmetry on brucellosis is key in designing pathways of intervention and response to the disease. This might be possible through efforts to optimise the health of humans, animals and the environment conceptualised as the One Health approach. Indeed, One Health is any societal added value in terms of the health of humans and animals, financial savings or environmental services, which is achievable by the cooperation of humans and veterinary medicines when compared to the two sectors working in silo (Zinsstag et al. 2015a). In this context, One Health is a problem-solving concept combining research and public health action in an iterative process of knowledge sharing (Zinsstag et al. 2015b), including with women. Studying the society and the way people behave is essential to understand human attitudes towards domesticated animals, their diseases and complexities affecting behaviours like culture, religion, age group, occupation and gender (Whittaker et al. 2015). Discussing narratives on the contributions of gender to the One Health concept can contribute to improve system thinking, participation, action and mutual learning in the co-production of knowledge for sustainable brucellosis control.

Knowing that women face barriers to access information on zoonotic diseases in many pastoral communities, this study also builds on the theoretical framework of social exclusion (Silver 1994) in accessing knowledge or information. The theory presents concepts of solidarity, specialisation and monopoly. The concept of solidarity is presented as a factor of exclusion through mutual support between members belonging to the same group, at the national, racial, ethnic and/or cultural level. The concept of specialisation identifies the social closure of members based on their respective professional aspects, the main source of social exclusion, because of their own rules and ways of acting that differ from other groups. Within the concept of monopoly, interactions based on the hierarchisation of powers place social actors in dominant and dominated social positions that perpetuate inequalities and promote social exclusion. Thus, this paper aims to describe mechanisms of knowledge production and transfer on brucellosis according to gender and specialisation, by assessing the way knowledge affects behaviours of pastoral communities for disease control.

## Methods

### Study area

**Fig. 1:**
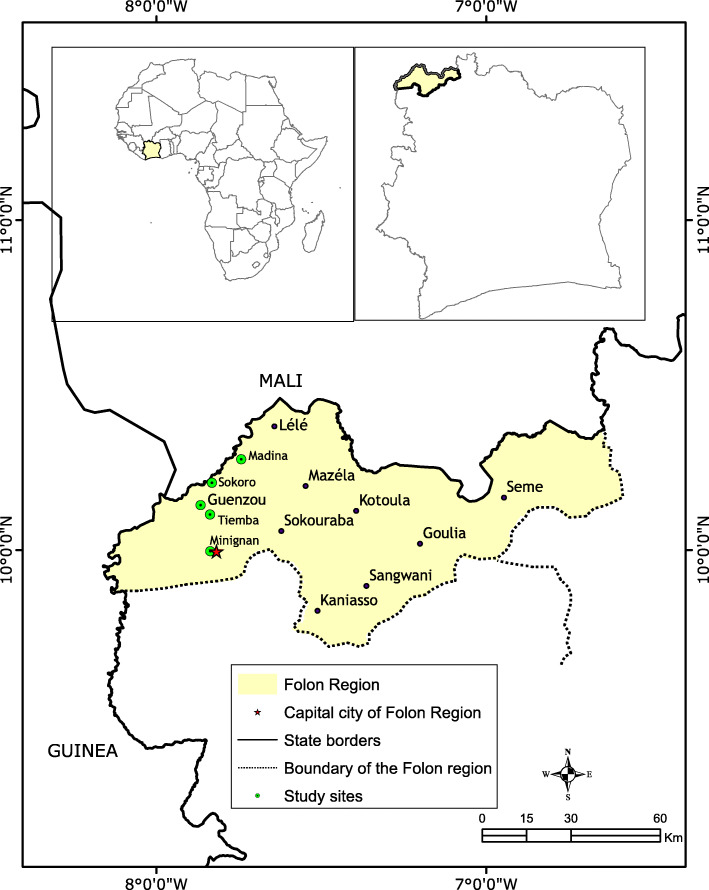
Map of the Folon region indicating study sites

The study was conducted in Minignan and Sokoro districts in the Folon region, in north-west Côte d’Ivoire, close to the border with Mali and Guinea (Fig. 1). This region situated in the sub-Sudanese savannah is part of the new area of expansion of pastoralism in West Africa. In Minignan, selected villages were Minignan [9° 59′38, 2668″ N (North); 7° 50′7, 2546″ W (West)], Gouenzou (10° 6′34, 88292″ N; 7° 50′14, 69652″ W) and Tiemba (10° 8′21, 63012″ N; 7° 51′1, 94508″ W). In the Sokoro district, they were Sokoro (10° 12′53, 86104″ N; 7° 50′21, 51672″ W) and Madina, usually called Marina, (10°16′28, 01676″ N; 7°44′34, 6704″ W). All selected villages were agro-pastoral areas and transhumance corridors. The Minignan and Sokoro districts recorded 20.039 cattle including 408 herds in transhumance in 2018 (Ministry of Animal and Fisheries Resources department of Folon region, annual report 2017-2018).

### Study design and sampling procedure

A community cross-sectional study was designed and conducted from July 2019 to February 2020. Inclusion criteria to participate in the study were the current or past involvement in pastoral activities for men and living with a livestock owner or shepherd for women. Participation was subject to a written informed consent.

### Sample size

This study is part of a research project involving epidemiologists, veterinarians and social scientists. For increased consistency and comparison purposes, we have used the same population and sample for the whole project. This sample size was estimated based on the comparison of two proportions at a confidence level of 95% and a power of 80% following the formula: *n* = [(*Z*_*α*/2_ + *Z*_*β*_)^2^ × (*P*_1_(1−*P*_1_) + *P*_2_(1−*P*_2_))]/(*P*_1_−*P*_2_)^2^, where *Z*_*α*/2_ is the critical value from the standard normal distribution (i.e. *Z*_*α*/2_ = 1.96 for confidence level of 95%) and *Z*_*β*_ is the critical value from the standard normal distribution giving *β* = 20% of the upper tail, i.e. 80% power (*Z*_0.8_ = 0.84). *P*_1_ and *P*_2_ are the expected sample proportions of exposure in diseased and non-diseased groups, respectively. In Côte d’Ivoire, assuming a prevalence of 1% of the disease in the non-exposed group and an expected prevalence of 5.3% in the exposed group, the required sample size is *n* = 256. To account for potential confounding for multivariate logistic analysis, the sample size will be increased by 20%, resulting in a total required sample size of *n* = 308. The exposed group was defined as individuals who have contact with animals and animal products in their daily work.

Thus, using this formula, a total of 252 participants from the pastoral community at the field site were required. This total was obtained after extrapolating a 1% rate of brucellosis in the unexposed group and an expected prevalence of 5.3% in the exposed group. In order to account for potential refusals and incomplete responses for the analysis, the sample size was increased by 20%. This resulted in a final estimated sample size of 308.

### Data collection process

The snowball method was used to select and interview livestock owners and shepherds, as well as their wives. The study targeted both transhumant and sedentary people. Sedentary people were met at home and/or at the paddock. Wives were met at home after written informed consent was given both by themselves and their husbands. In the case of a polygynous family, only one wife in the household was surveyed. Transhumant pastoralists were met at Sokoro on the Côte d’Ivoire-Mali border, whilst they were waiting to receive authorisation from a veterinarian to cross the border with their herds.

Mixed methods were used for data collection. The qualitative data collection was carried out first, making use of semi-structured interviews and focus group discussions (FGDs). A total of 26 semi-structured interviews were conducted with eight livestock owners, four shepherds, and a further five with livestock owners’ wives, and five with shepherd wives. A One Health approach was applied by jointly discussing animal and public health workers and their respective roles in knowledge production and transfer to pastoral communities. Four semi-structured interviews were conducted with health workers, one with a male nurse, another with a physician and two with veterinarians. Eight FGD were conducted with six people per group. Three focus groups were organised with sedentary livestock owners, whilst two discussion groups involved transhumant pastoralists, and participants of the three other FGD comprised respectively herdsmen, livestock owners’ wives and herdsmen’ wives. Printed pictures of hygromas and orchitis in ruminants were used during those interviews to structure the discussion on symptoms of brucellosis in animals. All interviews were recorded using a digital voice recorder. For the quantitative data collection, 320 questionnaires were completed. Interviews and questionnaires were prepared and conducted in Malinké (the local language commonly called *Dioula*), by a trained research assistant under a close supervision of the main researcher to ensure the accuracy of the translation of each discussion to French. The answers were then analysed and translated from French to English in this manuscript. Names and expressions from pastoral communities in local languages used to identify brucellosis symptoms were called Popular Nosological Entities (PNE). During the various interviews, brucellosis in animals was presented by the interviewer through its clinical symptoms such as hygromas, orchitis, drop in milk production and spontaneous abortions. Participants were also asked if they were familiar with the term brucellosis to describe this disease during the quantitative survey.

### Data management and analysis

Semi-structured interviews and FGDs were recorded with a voice recorder and transcribed into Microsoft Word (Microsoft Corporation, Redmond, WA, USA). Using MAXQDA 2018 software (VERBI GmbH, Berlin, Germany), information on the transcribed text was then grouped according to pre-established codes based on the interview guide and key covariates used for the study. After the first coding, data were recorded for further content analysis. Based on sources of information, a knowledge/information typology of production and transfer was defined by endogenous and exogenous routes. Indeed, endogenous communication occurs when a natural understanding emerges between individuals who are in contact. Exogenous communication entails the existence of an external device for the transmission of information (García-Gallego et al. 2013).

Quantitative data were collected by completing questionnaires on an Open Data Kit (ODK) application, stored in a database at the Centre Suisse de Recherches Scientifiques en Côte d’Ivoire on Microsoft Excel (Microsoft Corporation, Redmond, WA, USA). The data was imported into Statistical Package for the Social Scientists (SPSS) software (IBM SPSS statistics 20), to measure indicators and carry out statistical analyses like chi-square (*χ*^2^) comparison tests and Fisher/Pearson tests. To assess the link between different variables, the coefficient of difference between variables was significant when *p* ˂ 0.05. We compared variables between men and women using three main indicators: (1) their level of involvement on animal-keeping activities, (2) their level of information on brucellosis and (3) the risk of exposure to brucellosis related to raw milk consumption and handling animal products with their bare hands.

## Results

This section is structured around the socio-demographic characteristics and common illnesses in humans and animals, gendered knowledge on brucellosis, knowledge transfer on brucellosis transmission routes in communities, and brucellosis transmission risk practices among men and women in pastoral communities in the Folon region.

### Socio-demographic characteristics and common illnesses in humans and animals

A total of 320 people were enrolled and consented to participate to the study. Fulani (65.6%) and Malinké (34.4%) (Table 1) were the ethnic groups represented in the sample. Men were more educated, with a level of education ranging from primary school to university, when compared to women (41.7% vs 25.7%; *p* = 0.005, *p* < 0.05). Likewise, men were more engaged in livestock-keeping activities compared to women (97.2% vs 26.6%, *p* = 0.001 ˂ 0.05). Common human illness symptoms cited by respondents were fever (80%), general pains (54.7%), headache (49.1%) and diarrhoea (10.6%). In animals, frequent diseases mentioned were trypanosomiasis commonly known as *soumaya* or *soumayafi* meaning animal malaria (59.7%), followed by contagious bovine pulmonary disease (CBPP) called *Djôfô* (43.7%) and foot and mouth disease commonly called *Safa* (39.4%).

**Table 1:** Table of study population and sociodemographic features

	Total	Men (%)	Women (%)	*P*-value
Participants features				
**Total**	334	211 (65.9)	109 (34.1)	
**Herdsmen**	93	91 (43.1)	02 (1.8)	< 0.001*
**Livestock owners**	124	120 (56.9)	04 (3.7)	< 0.001*
**Livestock owners wives**	65	NA	65 (59.6)	NA
**Herdsmen wives**	36	NA	36 (33.0)	NA
**Educated**	116	88 (41.7)	28 (25.7)	**0.005**
**Engaged in livestock activities**	234	205 (97.2)	29 (26.6)	< **0.001**

Statistically significant (< 0.05) are highlighted in bold

*NA* Not applicable

*P-value was calculated according to Fisher’s exact tests

### Gendered knowledge on brucellosis

Knowledge of brucellosis was gender-oriented. Only 6.6% (*p* = 0.001 < 0.05) of men stated that they had heard about brucellosis. This was mostly from veterinarians during their routine activities (42.8%) (treatments and vaccination) and during training sessions (28.6%) with animal health workers. Women did not interact with animal health personnel and had not heard about brucellosis at health facilities where they often interact with human health staff. Interviews conducted with nurses, laboratory technicians and medical doctors in the study region showed that brucellosis was not listed among reported diseases. Brucellosis was barely known by human health personnel, diagnosis and treatment were not offered to patients. There is a differentiated access to information according to gender, but also according to the professional status of animal and human health workers. For human health specialists,

It is true; all of us [physicians] have little information about the One Health approach including zoonotic diseases such as brucellosis. But there is no opportunity; we cannot go deeply [into diagnosis]. We are really limited… (a physician, male, in Minignan).

Brucellosis is not part of the diseases under our epidemiological surveillance. So, we have never diagnosed a case of this disease (brucellosis) (a male nurse, in Minignan).

Knowledge on brucellosis is provided by animal health workers who target more men compared to women.

### Symptom-based knowledge of brucellosis

This study identified terms used by communities in their local language to identify brucellosis symptoms. These words in the local language were PNE associated with the symptoms of brucellosis which exists in that community. The PNE associated with brucellosis were usually in Malinke and Fulani languages. All PNE associated with brucellosis symptoms in that population were symptomatic descriptions in their local language. This meant that PC had some knowledge about brucellosis symptoms which, furthermore, could not be assigned to the disease as presented in Table 2.

**Table 2:** Associated Popular Nosological Entities (PNE) of major brucellosis symptoms in animals and their associated causes

Brucellosis symptoms	Malinké	Fulani	Local meaning	Associated causes
Hygromas	*Cégroubadimi Gbinningroudimi*	*Bakaalè* *N’Diam*	Knee pain/swollen knees *N’Diam* (water)	Aggravated trypanosomiasis Foot and mouth disease
Orchitis	*Bèlèkilifounou* *Kilifounou* *Tchèfounou* *côclifounou*	*Yèrèboutchi* or *Yèrèfondè*	Swollen testicles *Tchèfounou* (male organs swollen)	Ignored causes, no disease associated
Abortions	*connon-fili* *connon-tchan*	*Gnefitch or Fitch*	Aborted foetus	Trypanosomiasis, foot and mouth diseases, tick-borne diseases, dirty water, hunger during the dry season
Drop in milk production	*Nonnon dôgôya*	*Kossan madjè-timi*	Drop in milk production	Trypanosomiasis, pregnancy

### Knowledge transfer on brucellosis transmission routes in communities

Knowledge of brucellosis transmission routes, between animals and from animals to humans, was shown to pass from animal health staff to men in pastoral communities, and from there between men within the communities. Women had limited access to information on the disease. Among men who heard about the disease, 85.3% knew that brucellosis could be transmitted from infected animals to healthy animals and that this could happen particularly in cattle and sheep. However, the fact that people could be infected due to their contact with animals and animal products was not well known. More than half (57.2%) of those who had heard about brucellosis did not know whether the disease could be transmitted to humans. For those who knew that brucellosis is a zoonosis, drinking raw milk (60%) and eating undercooked meat (40%) were the main risk factors reported by participants for humans to be infected with the bacteria.

### Intention to comply with vaccination and slaughter of infected animals

We assessed the intention of compliance of pastoral communities regarding vaccination of their animals against brucellosis. Whilst individuals were eager to accept the vaccine (91.9%), its efficacy, availability and affordability were the main barriers to uptake. The intention to comply with a vaccination programme to prevent brucellosis was gender-oriented. Men were more likely to accept the vaccine compared to women (97.2% vs 81.7%; *p* < 0.001).

When it came to having to decide whether to slaughter infected animals as a control measure to prevent the transmission of brucellosis, there was a tendency (67.5%) among the participants to comply with this measure. However, the intention to comply with slaughtering infected animals was associated with compensation. We did not find any significant difference in acceptance concerning this control measure between men and women (67.7% vs 66.9%; *p* > 0.05).

### Transfer of endogenous and exogenous information on brucellosis within PC

Knowledge on brucellosis and/or its symptoms is acquired and transferred by both endogenous (from members of the pastoral community) and exogenous routes (from animal health workers). The endogenous route based on local knowledge is characterised by two forms of knowledge production and transfer on symptoms: firstly, through parental education, and secondly, through peers. Parental education involves young people learning from their parents during daily chores. The transfer of knowledge in that case follows generally the male line, from father to son as asserted by a livestock owner in Tiemba:

We started from childhood. Our dad taught us how to take care of cattle up to now. Even names of diseases like *bakaalè,* were taught by our fathers. (one male 48-year-old participant, from FGD with livestock owners in Tiemba)

In the endogenous route, the acquisition of knowledge on animal diseases takes place in the paddock and/or the fields where cattle graze most of the time. Those spaces are the schools for pastoral knowledge transmission, as shown in the following quote:

We learned in the bush… if an ox is sick, our dad often came to attend to the animal. While treating the animal he (dad) tells us the name of the disease and how to handle it. (one male 55-year-old participant, from FGD, with livestock owners in Tiemba)

The second way to acquire information on brucellosis among PC via the endogenous route was through peers. People from the same socio-professional group learn from each other. Information on cattle prices, grazing areas, transhumance routes, animal diseases, therapeutic itineraries, reputed veterinary doctors, etc., is shared among pastoral community members, especially by people belonging to the same professional sub-group such as livestock owners and shepherds. Members of those professional groups were usually of the same sex because of the social division of roles between men and women in the region. In both cases, the knowledge transferred relates to the identification of PNE such as *bakaalè*, *connon-fili* and health-seeking practices for animals. Interaction between people within the pastoral community provides opportunities to share advice or techniques on how to handle animal health problems, such as breaking open hygromas with a heated iron.

The exogenous route is based on socio-technical and scientific knowledge. Knowledge is transferred also through the male line. People targeted in this case were livestock keepers, shepherds and other actors such as butchers who are also generally animal keepers, of the information chain through training from extensionists or veterinarians. Public health workers were not stakeholders in this process. Among participants, only five informants, among the few who had heard about brucellosis, acquired the knowledge from veterinarians during their routine visits, and four during training sessions with animal health workers.

### Brucellosis transmission risk practices among men and women in pastoral communities

Men and women from pastoral communities in north Côte d’Ivoire exposure to brucellosis are centred around raw milk consumption, handling animals and disease symptom management. Most of the community members (66.6%) consume raw milk, regardless of gender. This accounted for 61.5% in women and 69.2% in men (*p* = 0.165 > 0.05). Their consumption of raw milk is related to several reasons including habits (59.4%), perceived flavour (40%) and richness in micro-nutrients, and lack of fire to cook their meals whilst in paddocks as presented in the following quotes. According to a member of the pastoral community, “Raw milk is good. It does not matter if you boil it. But when you do, it does no longer have strength [vitamins and nutrients]. It has less vitamins when you boil it” (a 41-year-old male shepherd in Minignan)

A participant during a group discussion describes what could be considered as milk benefits in the human body: “Whether it’s curdled milk, unboiled milk or boiled, I drink whatever I find. When I feel angry, and I drink a good cup of milk, I get really relaxed. When you do manual labour, and you drink milk, your performance increase and you can work a lot” (a 37-year-old female participant to FGD of livestock owners’ wives in Sokoro).

In addition, raw milk from cows with hygromas was still consumed in the region. Indeed, 8.4% of respondents, mainly men, had this habit compared to women (96.3% vs 3.7%, *p* < 0.001).

If cows have *bakaalè*, we can drink their milk without any problem … we can drink straight away without boiling it (a 29-year-old male participant to FGD with livestock owners in Gouenzou)

From our observations, raw milk from cows with hygromas is regularly sold in markets. Indeed, 11.6% of respondents sold milk from cows with hygromas, even without boiling it. Beyond practices linked to milk, there is also the risk in the management of animals with brucellosis symptoms. Some animal handling practices that pose a risk for transmission of brucellosis from animals to humans are presented in Table 3.

**Table 3:** Pastoral community behavioural risk factors of brucellosis transmission linked to livestock management

PC practices	Men, ***N*** (%)	Women, ***N*** (%)	***p***-value
Assist with bare hands during parturition	63 (19.7)	3 (22.2)	0.01
Using protective equipment or do not assist during parturition	148 (80.3)	106 (77.8)	
Handle aborted foetus with bare hands	106 (50.3)	7 (6.4)	0.00
Use of protective equipment or do not handle aborted foetus	105 (49.7)	102 (93.6)	
Handle placenta with bare hands	31 (14.7)	6 (5.5)	0.01
Use of protective equipment or do not handle placenta	180 (85.3)	103 (94.5)	
Break hygromas with bare hands	68 (32.3)	00 (0)	0.00
Use of protective equipment or do not break hygromas	143 (67.7)	109 (100)	

Table 3 raises the fact that men were more engaged than women in risk practices of brucellosis especially when handling animal discharge materials.

## Discussion

Brucellosis is a zoonotic disease with high prevalence in humans and animals in north Côte d’Ivoire (Kanouté et al. 2017). The aim of the present study was to describe mechanisms of co-production and the transfer of knowledge on brucellosis according to gender and professional specialisation in order to assess the way information can affect practices and transmission risk among pastoral communities. From the findings, there are three points highlighted for discussion. They are about masculinisation of animal-keeping practices, gendered asymmetry of knowledge production and transfer, and practices increasing the risk of transmission.

Findings from this study show a masculinisation of animal-keeping practices through a gendered division of labour in pastoral communities. The low level of involvement of women compared to men in the management of animals in the Folon region is common in Zimbabwe, Uganda and South Africa (Mupawaenda et al. 2009; Oluka et al. 2005; Njuki and Sanginga 2013). Labour division in the pastoralist context is a social reality in several places although it is gender related. However, women in many communities are involved in other activities like keeping small ruminants and processing dairy products that expose them to zoonotic diseases, similar to men.

Our study showed brucellosis is generally not well known among PC, and men are more likely to hear about the disease than women. This situation in north-west Côte d’Ivoire is similar to that observed in the district of Kilombero in Tanzania where only 7.2% of people had heard about the disease and where being male and more educated were predictors of knowledge of the disease (Mburu et al. 2021a). These findings from East to West Africa reveal women have limited access to information on brucellosis despite their exposure to the disease. It appears women are side-lined in the knowledge transfer process regarding brucellosis. As a result, women face more challenges than men when it comes to veterinary healthcare, accessing veterinary services and disease information (Galiè et al. 2017).

The gendered asymmetry on information on brucellosis shows gender inequality regarding public health aspects and sensitisation for brucellosis control. Indeed, gender inequalities present differences between men and women situations in a given field, based on a different perception of female and male social roles (Andrianjaka et al. 2001). Women are perceived to be better suited for housework and child rearing and, in the agro-pastoralist context, feeding small ruminants and processing dairy products. Thus, perceptions that a woman’s place is at home and not in the decision-making about livestock impact their access to information on diseases, including brucellosis. Moreover, the low level of education of women compared to men increases this asymmetry of access to information on brucellosis. Not understanding or speaking French, the language often used for health information dissemination, hinders their access to training on brucellosis. This link between female education and poor access to information on health matters, and the possibility of deciding for their health, is also a social reality in south-west Côte d’Ivoire (Adjamagbo and Guillaume 2001).

In addition, endogenous and exogenous sources of information are different channels for knowledge acquisition and dissemination on brucellosis in the Folon region. However, these different sources of information on brucellosis are similar to those in Tajikistan and Uganda, where people who mostly heard about brucellosis through friends or relatives are men (Lindahl et al. 2015; Kansiime et al. 2014). This contrasts with Jordan, where a large majority of participants were aware of brucellosis and acquired knowledge from official sources rather than by word of mouth among their peer/professional group (Guitian et al. 2015). Thus, knowledge transfer on brucellosis is still inter-personal in some areas, with the possibility of distortion during the diffusion process. There is a need to go beyond this inter-personal sensitisation by using media like the radio, broadcasts, social media, cartoons and movies, with well-informed health workers, in order to increase overall awareness of the disease.

Constituting transmission risks for brucellosis in humans includes handling hygromas and assisting parturition with bare hands. Such practices are common among men, whilst raw milk consumption by women and men among PC is also common practice (Tialla et al. 2015; Hundal et al. 2016; Muturi et al. 2018). The lack of information on brucellosis is a social and sanitary reality in the Folon region and associated with transmission risk for brucellosis, as demonstrated in a study on risk factors of brucellosis among abattoir personnel and pregnant women in North Cameroon (Awah-Ndukum et al. 2018). Based on scientific evidence, exposure to human brucellosis among pastoral populations is linked to gender, socio-professional activities and the level of information available or disseminated on brucellosis. Men and women in pastoral systems are doubly exposed to brucellosis in different ways. Women in north Côte d’Ivoire are exposed to brucellosis through raw milk processing and consumption whilst they lack information on the disease. Similarly, men are exposed through raw milk consumption and providing care to animals with bare hands. The remoteness of women in daily farming practices due to social distribution of roles is therefore a social factor in reducing exposure to brucellosis among women. These different social roles of men and women explain the fact that in several regions of the world, men are more often infected with brucellosis than women (Roushan et al. 2004; Bonfoh et al. 2012; Mohsenpour et al. 2015). Our findings show that men are more involved in practices risking brucellosis transmission during livestock management activities despite their exposure to information on brucellosis.

Contrary to expectations, access to information is not always positively associated with preventive measures. It was striking to notice that men developed the same at-risk practices, if not more, when compared to women. In addition, raw milk from cows with hygromas sold in markets in the region exposes consumers to brucellosis and other diseases transmitted by raw milk consumption. This risky trade in cow’s milk is a common social practice in certain regions of Africa (Bonfoh et al. 2003; Fokou et al. 2010; Kouame-Sina 2013). This is the reason why fermenting pasteurised milk should be promoted within pastoral communities as a means to control *Brucella abortus.*

It appears from this study that gendered management of livestock influences knowledge on brucellosis. This situation can jeopardise efforts for disease control. That is why efforts to optimise the health of humans, animals and the environment have been used for brucellosis control in Malta and Serbia through cooperation with public health and veterinary services, trans-disciplinarity, education and information sharing is needed (Buttigieg et al. 2018). This approach labelled as One Health is critically important for co-designing strategies for brucellosis control with the will from decision or policy-makers (Ghanbari et al. 2020).

This study focused only on gender knowledge aspects rather than whole parameters like comparison between transhumant and sedentary communities, veterinarians and public health workers which could provide more insight on the disease risk patterns. There is a need to investigate further public health positions in the prevention of brucellosis, knowledge transfer when seeking healthcare and the reasons why there is no talk between men and women about the disease.

## Conclusion and recommendations

Brucellosis is a priority zoonosis to control in Côte d’Ivoire where the main affected people are not well informed about the disease. Women, within pastoral communities in the north of the country, are less informed on brucellosis than men. Gender and social distribution of roles influence knowledge on brucellosis and maintain high-risk behaviours contributing to potentially contracting the disease. Persistence of risky practices related to the transmission of brucellosis among men, despite their better exposure to information on the disease compared to women, exposes the limitations of the existing knowledge transfer modes. It could be useful to further assess the various knowledge production and sharing processes to unpack the way various narratives are constructed and mobilised, to justify interventions and responses to zoonotic diseases. Transferring knowledge on brucellosis within the pastoral community including men and women is critically important. This could be possible using the One Health approach, with the collaboration of animal health and public health workers and considering environmental issues. Innovative strategies based on gender daily tasks such as safe dairy processing by women and safe animal husbandry to expand their herd for men can be the entry point for the prevention of brucellosis among pastoral communities. Such processes co-designed by actors from various sectors and categories, relying on indigenous and expert knowledge, are more likely to contribute in improving disease prevention and management efforts, whilst securing the livelihoods of pastoral communities.

## Data Availability

The datasets used and/or analysed during the current study are available from the corresponding author on reasonable request.
